# Prognostic Impact of Pulmonary Metastasectomy for Uterine Malignancies: A Retrospective Analysis of 38 Cases

**DOI:** 10.3390/cancers18010013

**Published:** 2025-12-19

**Authors:** Hiroyuki Adachi, Hiroyuki Ito, Takuya Nagashima, Tetsuya Isaka, Kotaro Murakami, Noritake Kikunishi, Naoko Shigeta, Aya Saito

**Affiliations:** 1 Department of Thoracic Surgery, Kanagawa Cancer Center, 2-3-2 Nakao, Asahi, Yokohama 241-8515, Japan; 2 Department of Surgery, Yokohama City University, Yokohama 236-0004, Japan

**Keywords:** pulmonary metastasectomy, uterine cancer, cervical cancer, cervix uteri, corpus uteri

## Abstract

Background: Uterine malignancies frequently metastasize to the lungs. Pulmonary metastasectomy has demonstrated survival benefits in some malignancies; however, its efficacy for uterine malignancies remains unclear. Methods: We retrospectively analyzed 38 patients who underwent pulmonary metastasectomy for uterine malignancies at the Kanagawa Cancer Center between 2010 and 2020. The primary endpoint was recurrence-free survival (RFS) after pulmonary resection. Results: The median patient age was 63 years. The primary sites were the cervical uteri (*n* = 22) and corpus uteri (*n* = 16). The FIGO stages at the time of treatment for the primary tumor were I, II, III, IV, and unknown in 20, 7, 9, 1, and 1 patient, respectively. The median disease-free interval (DFI), defined as the interval between primary treatment and first recurrence, was 26.5 months. Most patients had single metastasis (*n* = 32). The procedures for metastasectomy included lobectomy, segmentectomy, and wedge resection (*n* = 15, 8, and 15, respectively), and two cases resulted in microscopically incomplete resection. The median follow-up period after pulmonary metastasectomy was 57 months, with 16 patients experiencing recurrence after pulmonary metastasectomy (5-year RFS rate: 55.6%). Univariate analysis identified FIGO stage ≥ III, DFI < 12 months, presence of synchronous extrapulmonary recurrence, and uterine sarcoma as poor prognostic factors. No prognostic differences were found between cervical and corpus uteri cancers. Conclusions: Pulmonary metastasectomy may confer prognostic benefits in patients with uterine malignancies. Careful consideration is warranted for patients with advanced-stage primary tumors, early recurrence after primary treatment, synchronous extrapulmonary recurrence, and uterine sarcoma.

## Simple Summary

The efficacy of pulmonary metastasectomy for patients with uterine malignancies is uncertain. This study was conducted to evaluate the efficacy of pulmonary metastasectomy for patients with primarily treated uterine malignancies and recurrence with pulmonary metastases and showed good postoperative prognosis compared to patients who received chemotherapy ± immune checkpoint inhibitors. Considering our study’s findings and those of previous studies, pulmonary metastasectomy may confer prognostic benefits in patients with uterine malignancies, similar to those observed in patients with colorectal and renal cancers.

## 1. Introduction

The lung is one of the most metastasized organs from several types of malignancies [1,2,3,4,5]. Previously, distant metastatic lesions were treated with systemic therapy and surgical resection was not indicated. However, in 1995, Hellman and Weichselbaum proposed the concept of “oligometastases”, whereby metastatic lesions were found in only a limited number of regions [6]. Oligometastases is the state in which the patient shows distant relapses in only a limited number of regions; however, it includes the state with uncontrolled primary site having several distant metastases. In contrast, in 2008, Niibe et al. [7] proposed the concept of “oligo-recurrence”. Oligo-recurrence is a notion similar to oligometastases; however, according to Niibe et al. [8], the oligo-recurrence presents the condition of (i) one to several distant metastases/recurrences in one to several organs; (ii) primary site of the cancer controlled; (iii) one to several distant metastases/recurrences can be treated with local therapy; and (iv) no other distant metastases/recurrences other than those in (iii). In other words, oligo-recurrence is the state that all primary sites and metastatic sites can be controlled with local therapy, and many researchers have reported the efficacy of surgical resection for oligometastatic or oligo-recurrent lesions [9,10,11]. Based on the results, per some Japanese and American guidelines, pulmonary metastasectomy (PM) is recommended for appropriate cases for several kinds of malignancies, including colorectal cancer and renal cancer [12,13].

The lung is the most common distant metastatic organ in patients with uterine malignancies [14,15]; however, the efficacy of PM in patients with uterine malignancies remains uncertain [16,17]. Therefore, we aimed to conduct this retrospective study to evaluate the efficacy of PM in patients with primary uterine malignancies who experienced recurrence of lung metastases. We also explored the preoperative factors indicating poor postoperative survival after PM to identify patients who were not recommended to undergo the procedure.

## 2. Materials and Methods

### 2.1. Patients

We retrospectively reviewed the medical records of 38 patients with 39 lesions who underwent lung resection with curative intent for metastatic uterine malignancies at Kanagawa Cancer Center between January 2010 and December 2020. We usually select patients for the indication of PM according to the definition of “oligo-recurrence” proposed by Niibe et al. [8]. Thus, in this study, all primary tumors had been pathologically diagnosed as uterine malignancies and treated with local therapy (± chemotherapy) with curative intent before the PM, and all lung metastatic lesions were identified as oligo-recurrence. Post-treatment follow-up after the primary treatment was performed by gynecologists at our institute, and recurrence was confirmed when gynecologists detected suspicious findings on radiographic examination or echography, irrespective of the pathological confirmation. The study protocol followed the Japanese Ethical Guidelines for Epidemiological Studies and The Code of Ethics of the World Medical Association Declaration of Helsinki. The study was approved by the Institutional Review Board of Kanagawa Cancer Center (No. 2022EKI-74), and the requirement for informed consent was waived owing to the retrospective nature of the study.

### 2.2. Outcome Measures

We reviewed the patients’ medical records and accumulated the following clinicopathological variables: age, body mass index, comorbidities, the site of primary tumor, histological type of primary tumor, International Federation of Gynecology and Obstetrics (FIGO) staging [18] for primary tumor at the time of primary treatment, the modality of local therapy for primary tumor, disease-free interval (DFI), laterality of lung metastatic lesion, maximum diameter of the lung metastases, number of lung metastatic lesions, the first recurrent site after primary treatment, presence or absence of synchronous extrapulmonary metastases at the time of PM, presence or absence of conducting positron-emission tomography (PET) before PM, presence or absence of introducing systemic therapy for recurrent lesions before PM, the extent of lung resection, and adjuvant therapy after PM. Continuous variables are presented as medians with ranges, whereas categorical variables are presented as numbers with percentages.

We divided the histological type of primary tumor and analyzed as two variables—typical/atypical and carcinoma/sarcoma. We also defined the “typical” histological type as squamous cell carcinoma arising in the cervix and endometrial carcinoma arising in the uterine body, and the others (e.g., squamous cell carcinoma arising in uterine body, neuroendocrine carcinoma) as “atypical”, because we have relatively many cases whose endometrial carcinoma arose in cervix and we were interested in the relation of it and lung metastases. The DFI was defined as the period between the date of completion of curative-intent treatment for the primary tumor and the date of detection of recurrence. Recurrence-free survival (RFS) was defined as the period between the date of lung resection and the date of detection of another recurrence or the date of death from any cause and was analyzed as the primary outcome of this study. Overall survival (OS) after PM was also analyzed; however, we considered it could not be appropriate for primary outcome of this study due to the insufficient number of events.

### 2.3. Statistical Analysis

Survival curves were estimated using the Kaplan–Meier method and compared between groups using the log-rank test. Hazard ratios (HR) and 95% confidence intervals (CIs) were calculated using a Cox proportional hazards model to analyze the influence of each variable on postoperative prognosis after PM. Multivariate analysis was not conducted because of the small number of events. All analyses were performed using IBM SPSS statistics ver. 26 (IBM Corp., New York, NY, USA), with *p*-value < 0.05 considered as statistically significant.

## 3. Results

The patient backgrounds are shown in Table 1 (the summary of 38 cases is shown in Table S1). The median age at the time of PM was 63 (range: 34–84) years. Regarding the primary site, 22 patients had tumors arising from the cervix and 16 patients had tumors arising from the uterine body. The histology of the primary tumor was carcinoma in 34 patients and sarcoma in four patients. Of them, 22 patients were classified into “typical” and 16 patients were classified into “atypical.” More than half of the patients were stage I, and approximately 2/3 of patients underwent surgical resection for the primary tumor.

Regarding lung metastatic lesions, approximately 85% of the patients had a single lung lesion as the first recurrent site after treatment for the primary tumor. Most patients did not have synchronous extrapulmonary recurrence and did not receive induction therapy for metastatic lesions before PM. Wedge resection was performed for 15 patients, while lobectomy was performed for 15 patients each. Complete resection was achieved in all patients except for two. There was no death within 30 days postoperatively. Nor was there postoperative complication graded ≥ 3 according to Clavien-Dindo classification.

**Figure 1:**
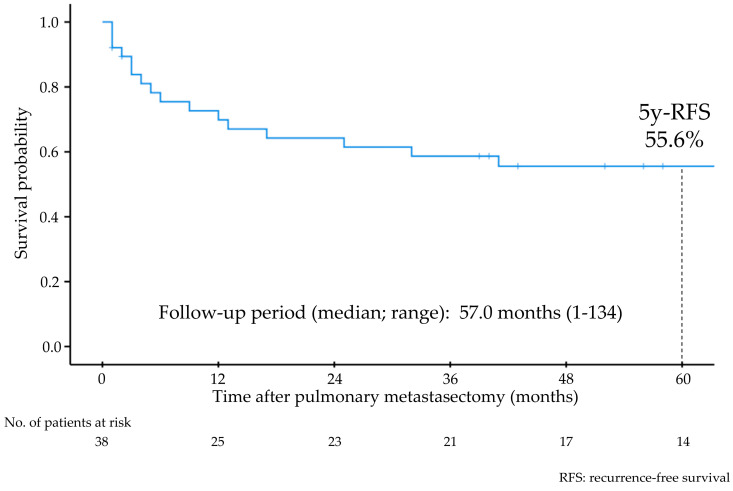
Recurrence-free survival of all patients after pulmonary metastasectomy.

**Figure 2:**
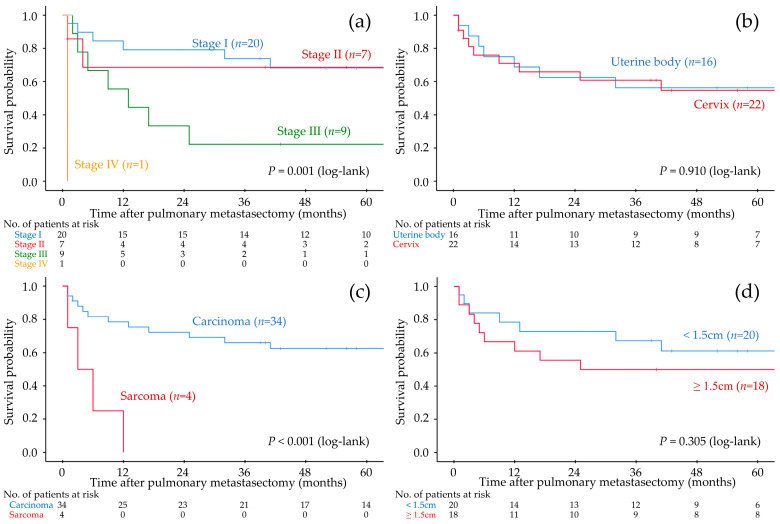
Recurrence-free survival after pulmonary metastasectomy stratified according to each variate. (**a**) FIGO stage of the primary tumor. (**b**) Origin of the primary tumor. (**c**) Histological type of the primary tumor. (**d**) Maximum diameter of lung metastases on computed tomography.

The median follow-up period after PM was 57 (range: 1–134) months, and 16 patients experienced recurrence after PM. The 5-year RFS rate and 5-year OS rate after PM were 55.6% (Figure 1) and 86.1%, respectively. Univariate analyses showed that stage III–IV primary tumors, sarcoma, DFI less than 12 months, and synchronous extrapulmonary recurrence were significant indicators of worse RFS (Figure 2, Table 2).

## 4. Discussion

Our study showed that the 5-year RFS rate after PM was 55.6% in patients who were primarily treated for uterine malignancies and developed lung metastases. We also revealed that stage III–IV disease at the time of primary treatment, sarcoma, DFI less than 12 months, and synchronous extrapulmonary recurrence were worse prognostic factors for RFS after PM.

The lung is the most frequent organ where metastases from primary uterine malignancies occur. Gardner et al. [16] conducted a retrospective study of data of patients who had stage IV primary uterine malignancies who were registered in the SEER database from 2010 to 2015. They reported that 62% of the 1241 patients with cancer of the corpus uteri and 59% of the 546 patients with cancer of the cervix uteri had lung metastases, and the 5-year disease-specific survival rate was 12% in patients with both cancer of the corpus uteri and cervix uteri. Ouldamer et al. [19] conducted a retrospective study on 1444 patients who underwent primary surgical treatment for early-stage cancer of the corpus uteri. In this study, 7.6% of patients experienced postoperative recurrence, and the most frequent recurrence site was the lungs (46.1% of all patients with recurrence). They also reported that the median DFI of patients who experienced only lung metastases was 9 months, and the 3-year OS (the period between surgical treatment for primary cancer and death from any cause) was only 50.6%. In addition, Ki et al. [20] reported that the prevalence of lung metastases in patients who underwent radical hysterectomy and postoperative concurrent chemoradiation/systemic chemotherapy was 3.6%, and the mean DFI of these patients was 12 months.

Lung metastasis is the most common recurrence and/or metastatic type of uterine malignancies. However, the prognosis of patients with lung metastases, which is equivalent to stage IV according to FIGO classification, is unsatisfactory. Recently, similar to other types of malignancies, the efficacy of immune checkpoint inhibitors (ICI) in the treatment of recurrent/progressive uterine malignancies has been reported. Makker et al. [21] reported the efficacy of lenvatinib plus pembrolizumab compared to that via chemotherapy for previously treated advanced cancer of the corpus uteri. They showed significantly improved results wherein median OS of patients who received lenvatinib + pembrolizumab therapy was 18.7 months compared to 12.2 months of patients who received chemotherapy. In addition, the study by O’Malley et al. [22] demonstrated more effective results with pembrolizumab in patients with cancer of the corpus uteri with high microsatellite instability. In these patients, the median progression-free survival after the introduction of pembrolizumab was 13.1 months (95% CI: 4.3–34.4 months). As for patients with cancer of the cervix uteri, Monk et al. [23] showed that 1st line pembrolizumab + chemotherapy significantly improved the OS of patients with recurrent/metastatic cancer of the cervix uteri compared to that via chemotherapy alone, especially in patients showing positive programmed death-ligand 1 (PD-L1) status (median OS for all patients: 26.4 vs. 16.8 months, for PD-L1 positive patients; 28.6 vs. 16.5 months). Thus, ICIs have improved the survival of patients with stage IV uterine malignancies; however, these results are still inferior to those of our study. However, comparing prognoses for patients with distant recurrence after local treatment with those for patients with stage IV de novo disease may need caution, because some researchers reported different prognoses of patients with postoperative distant recurrence and those of patients with stage IV de novo disease in some kinds of malignancies [24,25]

The results of previous studies support our findings (Table 3). Kanzaki et al. [26] conducted a retrospective study that included 57 patients who underwent PM for uterine malignancies and reported good postoperative survival (5-year RFS and 5-year OS after PM were 40.7% and 68.8%, respectively). Furthermore, Nobori et al. [27] reported good postoperative OS in a retrospective study conducted in the largest cohort of patients to date. As shown in Table 3, the postoperative survival of patients who underwent PM for uterine malignancies appeared to be better than that of patients treated with chemotherapy, even with ICIs. Thus, we believe that PM should be recommended for patients with uterine malignancies, similar to those with colorectal or renal cancer. According to previous and present studies, we consider that patients with a long DFI can be good candidates for PM for uterine malignancies, although the cutoff value of DFI (12 or 24 months) remains contradictory. However, the other variables that influence the selection of candidates for PM remain unclear. Kanzaki et al. [26] showed that primary tumor arising from the uterine body was a worse prognostic factor after PM, while Nobori et al. [27] showed that primary tumor arising from the cervix was a worse prognostic factor after PM. Our study showed that primary histology (sarcoma) was a worse prognostic factor after PM, while Paramanathan et al. [28] showed good postoperative prognosis after PM even if the patients had uterine sarcoma. Although some studies indicated that larger metastatic lesions were a worse prognostic indicator, the extent of lung resection did not influence postoperative prognosis in any study.

The strength of this study is that we found an early FIGO stage, including stage I and II, at the time of primary treatment was a significantly better prognostic factor after PM with estimating survival curve using the Kaplan–Meier method. To the best of our knowledge, no other previous studies have shown similar results. Adachi et al. [29] and Paik et al. [30] also evaluated the influence of primary FIGO stage on prognosis after PM using a Cox proportional hazards model; however, they did not show statistical difference between patients with stage I-II and patients with stage III-IV. It may be due to the small sample size of their study. Kanzaki et al. [26] also evaluated the influence of primary FIGO stage in their study, which was conducted among the largest cohort of patients to date. However, they divided patients into stage I group and stage II-IV group for some reason and showed no statistical difference between groups with use of a Cox proportional hazards model. In contrast, we found that the prognosis after PM in patients with stage I and those with stage II were estimated as equivalent, whereas those with stage III or stage IV were estimated worse than those with stage I and stage II, and consequently, early FIGO stage, including stage I and stage II, was a significantly better prognostic factor after PM with use of a Cox proportional hazards model. Thus, our study is the first showing that patients with FIGO stage II at the time of primary treatment can also be a good candidate for PM when they show the findings of oligo-recurrence. However, because these were all retrospective studies conducted in small patient cohorts, prospective randomized controlled trials or at least multicenter observational studies conducted with large patient cohorts are needed to resolve these issues.

Regarding the therapeutic modality for lung metastases, stereotactic body radiotherapy (SBRT) can be a treatment option. Although SBRT is recognized as a less-invasive therapy compared to surgical resection, Londero et al. [32] reported in their systematic review that PM seemed to guarantee better long-term survival compared to SBRT and concluded that PM remains the treatment of choice for lung oligometastases. In addition, it must be merited that pathological confirmation whether the lung lesions are truly metastases or not can be obtained if PM has been undergone. It should provide important data to select the next treatment strategy. Thus, we consider that PM, rather than SBRT, is effective and should be undergone if patients are surgically tolerable.

Our study has some limitations. First, our study had a small sample size, including only 38 patients and 16 events (recurrence after PM); thus, multivariate analyses could not be performed. Second, our study was conducted at a single institution, and we had no data regarding patients who did not undergo PM, even if they could be candidates. The selection of patients who consulted thoracic surgeons to undergo PM was completely up to gynecologists, which may have led to selection bias. To evaluate the efficacy of PM adequately, the comparison of patients who underwent PM and patients who were treated with chemotherapy alone should be needed. Despite these limitations, we believe that our study shows valuable findings and will influence the future treatment of patients with lung metastases from uterine malignancies, particularly given that it was conducted among the third-largest cohort of patients to date. However, prospective randomized controlled trials should be conducted to confirm the efficacy of PM in patients with uterine malignancies.

## 5. Conclusions

Similar to previous studies, we found that patients with recurrent uterine malignancies who underwent PM showed comparatively good postoperative survival. Similar to several types of malignancies, PM may confer prognostic benefits in patients with uterine malignancies. However, careful consideration should be warranted for patients with advanced FIGO stage at the time of primary treatment, with a shorter DFI, synchronous extrapulmonary recurrence, and uterine sarcoma. Multicenter observational studies conducted with large patient cohorts may provide data concerning precise patient selection, and prospective randomized controlled trials are needed to confirm the efficacy of PM for patients with lung metastases from uterine malignancies.

## Figures and Tables

**Table 1:** Patient background, treatment for lung metastases, and postoperative outcome.

Variables	
**Age** (years)	63 (34–84)
**Body mass index** (kg/m^2^)	
<18.5	5 (13.2)
18.5–24.9	25 (65.8)
≥25.0	8 (21.1)
**Comorbidities**	
None	30 (78.9)
Present	8 (21.1)
**Origin of primary site**	
Cervix	22 (57.9)
Uterine body	16 (42.1)
**Histological type (1)**	
Carcinoma	34 (89.5)
Sarcoma	4 (10.5)
**Histological type (2)**	
Typical ^1^	22 (57.9)
Atypical	16 (42.1)
**Primary stage** (FIGO)	
I	20 (52.7)
II	7 (18.4)
III	9 (23.7)
IV	1 (2.6)
Unknown	1 (2.6)
**Local therapy for primary tumor**	
Surgery	25 (65.8)
Radiotherapy	13 (34.2)
**DFI**^2^ (months)	26.5 (1–163)
**Laterality of lung metastases**	
Right	19 (50.0)
Left	18 (47.4)
Bilateral	1 (2.6)
**Maximum diameter of the lung metastases** (cm)	1.4 (0.5–7.0)
**Number of lung metastatic lesions**	
1	32 (84.2)
2	5 (13.2)
≥3	1 (2.6)
**First recurrent site after primary treatment**	
Lung	33 (86.8)
Others	4 (10.5)
Unknown	1 (2.6)
**Synchronous extrapulmonary recurrence**	
None	35 (92.1)
Present	3 (7.9)
**PET before pulmonary metastasectomy**	
None	2 (5.3)
Performed	36 (94.7)
**Systemic therapy for recurrent lesions before pulmonary metastasectomy**	
None	31 (81.6)
Performed	6 (15.8)
Unknown	1 (2.6)
**Extent of lung resection**	
Wedge resection	15 (39.5)
Segmentectomy	8 (21.0)
Lobectomy	15 (39.5)
**Complete resection**	
R0	36 (94.7)
R1	2 (5.3)
**Follow-up period after pulmonary metastasectomy** (months)	57 (1–134)
**Adjuvant chemotherapy after pulmonary metastasectomy**	
None	23 (60.6)
Performed	14 (36.8)
Unknown	1 (2.6)
**Recurrence after pulmonary metastasectomy**	
None	22 (57.9)
Present	16 (42.1)
**Outcome after pulmonary metastasectomy**	
Alive	32 (84.2)
Death of other disease	1 (2.6)
Cancer death	5 (13.2)

^1.^ Including squamous cell carcinoma that arose from the cervix and endometrial carcinoma that arose from the uterine body. ^2.^ The period between the date of completing curative therapy for the primary tumor and that of the detection of recurrence. DFI: disease-free interval; FIGO: International Federation of Gynecology and Obstetrics; PET: positron emission tomography.

**Table 2:** Univariate analyses for recurrence-free survival after pulmonary metastasectomy.

		Univariate		
**Variable**		**HR**	**95% CI**	***p*-Value**
**Age** (years)	<60	1		0.520
	≥60	1.415	0.491–4.078	
**Body mass index** (kg/m^2^)	<18.5	1		0.451
	≥18.5	0.451	0.127–1.594	
**Origin of primary site**	Cervix	1		0.911
	Uterine body	0.945	0.352–2.538	
**Histological type (1)**	Carcinoma	1		0.002 *
	Sarcoma	6.848	1.968–23.832	
**Histological type (2)**	Typical	1		0.718
	Atypical	1.200	0.446–3.228	
**Primary stage (FIGO)**	I, II	1		0.009 *
	III, IV	3.747	1.382–10.164	
**DFI (1)**	<12 months	1		0.010 *
	≥12 months	0.259	0.093–0.724	
**DFI (2)**	<24 months	1		0.716
	≥24 months	0.833	0.312–2.224	
**Maximum diameter of the** **lung metastases**	<1.5 cm	1		0.313
	≥1.5 cm	1.645	0.625–4.327	
**Number of lung metastatic** **lesions**	Single	1		0.799
	Multiple	1.177	0.335–4.134	
**First recurrent site after** **primary treatment**	Lung	1		0.931
	Others	0.955	0.334–2.726	
**Synchronous extrapulmonary recurrence**	None	1		0.002 *
	Present	8.554	2.156–33.942	
**Systemic therapy for recurrent lesions before pulmonary** **metastasectomy**	None/unknown	1		0.788
	Performed	1.136	0.448–2.883	
**Extent of lung resection**	Lobectomy	1		0.065
	Wedge/segmentectomy	3.271	0.929–11.510	
**Adjuvant chemotherapy after pulmonary metastasectomy**	None	1		0.085
	Performed	0.383	0.128–1.141	

* Statistically significant. CI: confidence interval; DFI: disease-free interval; FIGO: International Federation of Gynecology and Obstetrics; HR: hazard ratio.

**Table 3:** Reports published after 2010.

Author	Year	Study Period	*N*	5 Y RFS	5 Y OS	Factors Associated with Worse Prognosis [For OS/RFS]	
						Univariate	Multivariate
Paramanathan [14]	2013	2001–2011	13 (Sarcoma)	N/A	66.0%	N/A	N/A
Adachi [29]	2015	1985–2013	23	44.7%	81.7%	DFI < 24 months [OS]	N/A
Paik [30]	2015	1995–2011	29	N/A	48.2%	Presence of symptoms related to lung metastasis [OS] Number of lung metastasis > 3 [OS]	Presence of symptoms related to lung metastasis [OS] Number of lung metastasis > 3 [OS]
Anile [31]	2017	1997–2010	19	12.8%	40.9%	DFI < 24 months [OS] Recurrence after pulmonary metastasectomy [OS] Introduction of adjuvant therapy after initial treatment [OS]	DFI < 24 months [OS] Recurrence after pulmonary metastasectomy [OS]
Kanzaki [26]	2020	2006–2015	57	40.7%	68.8%	Location of the primary tumor (uterine body) [RFS] Size of metastatic tumor (>2 cm) [RFS] DFI ≤ 24 months [OS]	N/A
Nobori [27]	2023	1984–2016	319	N/A	66.5%	Primary site (cervix) [OS] Surgical approach (thoracotomy) [OS] Date of metastasectomy (1984–2000) [OS] DFI < 12 months [OS] Number of metastatic tumors ≥4 [OS]	Primary site (cervix) [OS] Number of metastatic tumors ≥ 4 [OS]
Present study	2025	2010–2020	38	55.6%	86.1%	Primary stage (III–IV) [RFS] Histological type (sarcoma) [RFS] DFI < 12 months [RFS] Synchronous extrapulmonary metastases (present) [RFS]	N/A

DFI: disease-free interval; N/A: not available; OS: overall survival; RFS: recurrence-free survival.

## Data Availability

The data presented in this study are available upon request from the corresponding author owing to the rules of our Institutional Review Board.

## Supplementary Materials

The following supporting information can be downloaded at: https://www.mdpi.com/article/10.3390/cancers18010013/s1, Table S1. Summary of 38 Cases that underwent lung resection with curative intent for metastatic uterine malignancies.

## Abbreviations

The following abbreviations are used in this manuscript:

CI	confidence interval
DFI	disease-free interval
HR	hazard ratio
N/A	not available
OS	overall survival
PD-L1	programmed death-ligand 1
PET	positron-emission tomography
PM	pulmonary metastasectomy
RFS	recurrence-free survival
SBRT	stereotactic body radiotherapy
