# Supplementary material for: Prognostic Impact of Pulmonary Metastasectomy for Uterine Malignancies: A Retrospective Analysis of 38 Cases
Source: Cancers (Basel). 2025 Dec 19;18(1):13. doi: 10.3390/cancers18010013 (PMC12784751; doi:10.3390/cancers18010013)
Supplement: Supplementary file 1 [file cancers-18-00013-s001.zip › cancers-4005725-supplementary.pdf]

cancers

MDPI

Table S1. Summary of 38 Cases who underwent lung resection with curative intent for metastatic uterine malignancies.

No.	Age	BMI	Comorbidity	Primary Site	Histological Type (1)	Histological Type (2) *	Primary Stage	Local Therapy	DFI	Laterality	Tumor Size	Number of Lung Meta	First Rec Site	Synchronous Extrapulmonary Rec	PET	Systemic Therapy for Rec before PM	Extent of PM	Complete Resection	Adjuvant Cx after PM	Rec after PM	RFS	Prognosis	OS
**1**	62	21.1	-	body	Endometrial	Typical	I	Op	19	right	1.4	1	Lung	-	+	-	Lob	R0	+	-	134	Alive	134
**2**	56	22.0	+	body	Endometrial	Typical	?	Op	34	left	1.9	1	?	-	+	?	Lob	R0	?	-	120	Alive	120
**3**	66	25.4	+	body	Endometrial	Typical	I	Op	12	left	1.4	1	Lung	-	+	-	Lob	R0	+	-	77	Alive	77
**4**	44	23.9	-	body	Endometrial	Typical	II	Op	18	left	2.7	2	Lung	-	+	-	Seg	R0	+	-	87	Alive	87
**5**	77	19.8	+	Cervix	Squamous	Typical	II	RT	26	left	3.6	1	Lung	-	+	-	Lob	R0	+	-	75	Death of other disease	75
**6**	34	36.9	-	Cervix	Endometrial	Atypical	I	Op	59	right	0.9	1	Lung	-	-	-	Wedge	R0	-	-	2	Alive	2
**7**	62	21.0	-	Cervix	AdSq	Atypical	I	Op	23	left	1.0	1	Lung	-	+	+	Wedge	R0	-	-	107	Alive	107
**8**	43	18.8	-	Cervix	Endometrial	Atypical	I	Op	39	left	0.7	1	Lung	-	+	-	Wedge	R0	-	-	94	Alive	94
**9**	51	18.3	-	Cervix	Neuro	Atypical	I	Op	54	left	2.4	1	Local →RT	-	+	-	Lob	R0	+	-	103	Alive	103
**10**	38	19.2	-	Cervix	Endometrial	Atypical	I	Op	13	right	1.2	2	Lung	-	+	-	Lob	R0	+	-	96	Alive	96
**11**	46	18.7	-	Cervix	AdSq	Atypical	I	Op	37	left	2.3	1	Lung	-	+	-	Lob	R0	+	-	73	Alive	73
**12**	80	25.6	+	body	Endometrial	Typical	I	Op	37	right	3.2	1	Lung	-	+	-	Lob	R0	-	-	65	Alive	65
**13**	80	22.2	+	body	Neuro	Atypical	I	Op	11	left	2.0	1	Lung	-	+	-	Lob	R0	-	-	85	Alive	85
**14**	70	43.8	+	body	Endometrial	Typical	I	Op	63	left	1.4	1	Urethra →Op	-	+	-	Wedge	R0	-	Lung	32	Alive	81
**15**	52	18.0	-	Cervix	AdSq	Atypical	III	RT	30	right	1.4	1	Lung	Local →Op (1M before PM)	+	-	Seg	R0	+	Para Ao LN	9	Alive	28
**16**	72	18.1	+	Cervix	Squamous	Typical	III	RT	74	right	1.7	3	Lung	-	+	+	Wedge	R0	-	Lung, Neck LN	25	Alive	67
**17**	35	17.7	-	Cervix	Squamous	Typical	IV	RT	6	left	4.8	2	Lung	paraAo LN → resected	+	+	Seg	R1	-	Disseminat ion	1	Cancer death	6

														with primary tumor (2M before PM)									
**18**	69	33.1	-	body	Sarcoma	Atypical	I	Op	17	right	4.1	1	Local →RT	-	+	+	Lob	R0	+	Liver	6	Cancer death	12
**19**	53	19.4	-	body	Endometrial	Typical	I	Op	15	left	1.3	1	Lung	-	+	+	Seg	R0	-	-	58	Alive	58
**20**	43	27.5	-	Cervix	Endometrial	Atypical	I	Op	48	left	0.8	1	Lung	-	+	-	Wedge	R0	-	-	39	Alive	39
**21**	78	22.1	-	Cervix	Squamous	Typical	II	RT	27	left	1.6	1	Lung	-	+	-	Seg	R0	-	Mediastinal LN	1	Alive	19
**22**	42	20.0	-	Cervix	Squamous	Typical	III	RT	11	right	1.3	1	Lung	Left neck LN →Op (2M after PM)	+	-	Wedge	R0	-	Neck LN	2	Cancer death	14
**23**	44	25.6	+	body	Endometrial	Typical	I	Op	14	left	0.5	1	Lung	-	+	-	Wedge	R0	+	-	72	Alive	72
**24**	66	24.1	-	body	Endometrial	Typical	I	Op	163	Bi-lateral	0.9	2	Incision site →Op	-	+	-	Wedge	R0	+	-	52	Alive	52
**25**	84	19.8	-	Cervix	Squamous	Typical	III	Op	7	right	1.0	1	Lung	-	+	-	Wedge	R0	-	-	43	Alive	43
**26**	64	20.9	-	Cervix	Squamous	Typical	II	RT	141	right	3.9	1	Lung	-	+	-	Lob	R0	-	-	40	Alive	40
**27**	67	24.0	-	Cervix	Endometrial	Atypical	I	Op	69	right	0.8	1	Lung	-	+	-	Wedge	R0	-	Lung	41	Alive	48
**28**	50	20.4	-	Cervix	Endometrial	Atypical	III	RT	1	right	0.7	1	Lung	-	-	-	Seg	R0	+	Lung	13	Alive	38
**29**	64	24.8	-	Cervix	Squamous	Typical	III	RT	7	right	7.0	1	Lung	-	+	-	Lob	R0	-	Mediastinal LN	3	Alive	36
**30**	70	23.8	-	body	Endometrial	Typical	III	Op	2	right	1.8	1	Lung	-	+	-	Seg	R0	-	Vaginal stump	5	Alive	30
**31**	68	21.2	-	body	Sarcoma	Atypical	I	Op	32	left	0.8	1	Lung	-	+	-	Wedge	R0	+	Lung	3	Alive	99
**32**	79	29.0	-	body	Endometrial	Typical	III	Op	39	right	1.9	1	Lung	-	+	-	Wedge	R0	-	Mediastinal LN	17	Alive	30

**33**	42	23.5	-	body	Sarcoma	Atypical	I	Op	3	right	1.0	1	Lung	-	+	-	Lob	R0	-	Dissemination	1	Cancer death	18
**34**	68	21.0	-	body	Sarcoma	Atypical	I	Op	32	right	1.5	2	Lung	-	+	+	Wedge	R0	-	Lung	12	Alive	58
**35**	64	21.5	-	Cervix	Squamous	Typical	II	RT	14	left	1.7	1	Lung	-	+	-	Seg	R0	-	Brain	4	Cancer death	8
**36**	57	17.3	-	Cervix	Endometrial	Atypical	II	RT	107	right	0.6	1	Lung	-	+	-	Lob	R0	-	-	1	Alive	1
**37**	60	22.2	-	Cervix	Squamous	Typical	III	RT	17	right	5.2	1	Lung	-	+	-	Lob	R0	-	-	64	Alive	64
**38**	78	25.6	-	Cervix	Squamous	Typical	II	RT	35	left	1.4	1	Lung	-	+	-	Wedge	R1	+	-	56	Alive	56

* “Typical” histological type was defined as squamous cell carcinoma arising in the cervix and endometrial carcinoma arising in the uterine body, and the others was classified into “atypical”.

Ao: aorta, BMI: Body mass index (kg/m2), Cx: chemotherapy, DFI: disease-free interval (the period between the date of completion of curative-intent treatment for the primary tumor and the date of detection of recurrence [months]), LN: lymph node, Lob: lobectomy, Meta: metastases, Neuro: neuro-endocrine carcinoma, Op: operation, OS: overall survival (the period between the date of lung resection and the date of death from any cause [months]), PET: positron-emission tomography, PM: pulmonary metastasectomy, Rec: recurrence, RFS: recurrence-free survival (the period between the date of lung resection and the date of detection of another recurrence or the date of death from any cause [months]), RT: radiotherapy, Seg: segmentectomy, Wedge: wedge resection.
